# Supplementary figures and images for: IIV-6 Inhibits NF-κB Responses in Drosophila
Source: Viruses. 2019 May 1;11(5):409. doi: 10.3390/v11050409 (PMC6563256; doi:10.3390/v11050409)

# *Drosomycin*

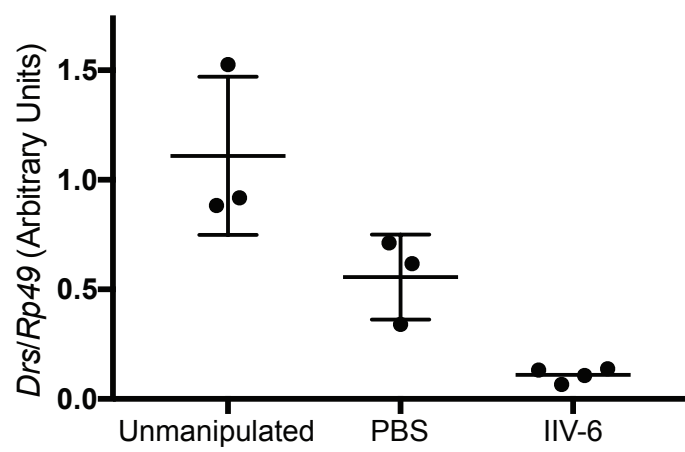

Supplement: Supplementary file 1 [file viruses-11-00409-s001.zip › Supplemental Figure 2.pdf]

*GAPDH1*

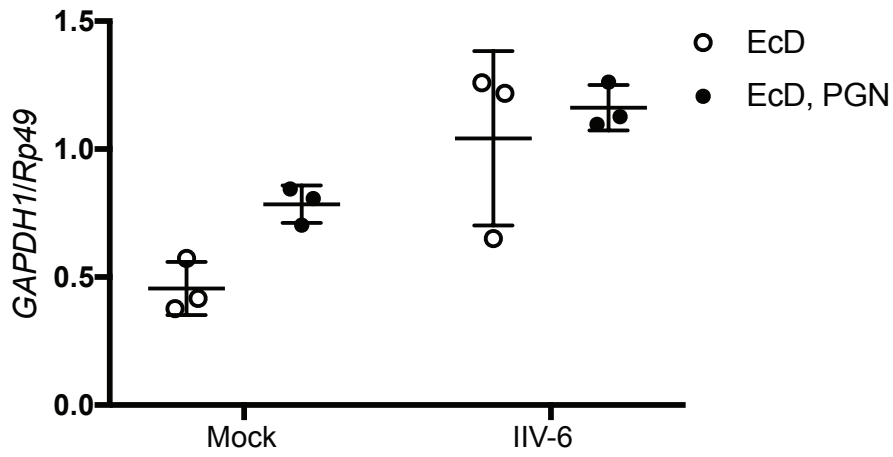

Supplement: Supplementary file 1 [file viruses-11-00409-s001.zip › Supplemental Figure 1.pdf]
